# Factors that influence the implementation of health and social care Standards: a systematic review and meta-summary protocol

**DOI:** 10.12688/hrbopenres.13212.1

**Published:** 2021-02-24

**Authors:** Yvonne Kelly, Niamh O’Rourke, Rachel Flynn, Josephine Hegarty, Laura O’Connor

**Affiliations:** 1 Health Information and Quality Authority, Unit 1301, Citygate, Mahon, Cork, T12 Y2XT, Ireland; 2 Catherine McAuley School of Nursing and Midwifery, Brookfield Health Sciences Complex, University College Cork, College Road Cork, T12 AK54, Ireland

**Keywords:** Standards, Implementation, Enablers, Barriers, Healthcare, Social care, Systematic review, Meta-summary

## Abstract

Health and social care Standards are evidence-based statements that demonstrate a desired level of care. Setting Standards for health and social care is a mechanism by which quality improvements can be achieved. Limited evidence exists on appropriate implementation strategies to overcome challenges with implementing Standards. In order to inform the design of implementation strategies, there is a need to examine factors that influence their implementation. The aim of this protocol is to set out a comprehensive plan to undertake a systematic search, appraisal and mixed research synthesis of the international literature that examines implementation of health and social care Standards.

A research question, “What are the enablers and barriers to implementing health and social care Standards in health and social care services?” was designed using the ‘SPICE’ (Setting, Perspectives, Interest phenomenon of, Comparison, Evaluation) framework. Electronic databases, grey literature and reference lists from included studies will be searched. Primary qualitative, quantitative descriptive and mixed methods studies reporting on enablers and barriers to implementing nationally endorsed Standards, will be included. The review will focus on experiences and perspectives from multi-level stakeholders including patient and public involvement. The quality of studies will be appraised using appropriate tools and findings used to weight interpretation of findings. Search outputs, data extraction and quality appraisal will be undertaken by two reviewers independently. Sandelowski meta-summary will be used to synthesise the data. Frequency and intensity effect sizes of enablers and barriers will be calculated to evaluate their prevalence across the studies. The Confidence in Evidence from Reviews of Qualitative research (CERQual) approach will be applied to assess confidence in the findings of the review.

Findings from this examination will inform influencing factors to implementation. Subsequently, this will contribute to pairing Standards with appropriate implementation strategies that will optimise the enabling factors and overcome challenges to implementation.

## Introduction and background

Health systems worldwide have led a continued quest to achieve patient safety in the delivery of care. International Standards-setting bodies develop and publish Standards for health and social care services as a quality improvement approach for patient safety. The World Health Organization (WHO) advocates setting Standards to act as leverages to improve the quality of care delivered in health and social care services ^1^. Standards comprise of statements describing a process or outcome of care. Key words used to define Standards by Standards-setting bodies include “evidence-based” ^2^, “high level outcomes” ^3^ and “level of performance.” ^4^ Setting Standards is an important means for shaping the behaviour of health and social care providers, health and social care professionals and other key stakeholders. The many benefits of their implementation include promoting a consistent level of care, quality assurance, and sharing an understanding of what quality, safe practice looks like for people using healthcare services and service providers ^3^. The Australian Commission on Safety and Quality in Healthcare reported significant improvements following implementation of the Preventing and Controlling Healthcare-Associated Infection Standard ^5^. Implementation of the management strategies within the Standard have reduced cases of *Staphylococcus aureus* infections, central-line associated bloodstream infections and have increased activities in antimicrobial stewardship programmes ^5^. The Standard-setting body in England, the National Institute for Health and Care Excellence (NICE) use shared learning case studies to show how implementing their quality standards have led to improvements in practice. One such case study describes how a care provider used five sets of NICE quality standards for social care to develop audit tools to assess and evaluate performances across 70 care homes ^6^. Consequently, “supporting people to live well with dementia” was an area that had been identified as needing improvement ^6, 7^. Findings from the National Audit of Intermediate Care in England reported that waiting times for service delivery in intermediate care and enablement services reached targets as recommended in NICE quality Standards, highlighting progress in the delivery of adult social care ^7^.

Differences exist among countries worldwide in approaches to implementing and monitoring health and social care Standards. Some countries, for example England ^2^ and Northern Ireland ^8^ develop Standards that describe optimum practices, and can be used as benchmarks to determine performance levels during inspections by their regulatory bodies. Inspection is a process where subject matter experts visit health and social care settings to assess or regulate a service’s conformance with nationally endorsed Standards ^9^. The Standard-setting body in Ireland, the Health Information and Quality Authority (HIQA) has a licensing regulatory framework for the monitoring, inspection and registration of residential care settings, against regulations and associated social care Standards ^3^. Accreditation is another approach where healthcare organisations are accredited according to pre-determined Standards ^9^. Accreditation in itself, can positively impact clinical performance, organisational culture and leadership ^10^. The Australian and American healthcare systems use independent accreditation agencies to monitor adherence to healthcare Standards ^4, 11^.

Standards are multi-faceted interventions including many evidence-based declarative statements relating to expected actions and behaviours that involve multiple stakeholders and multiple efforts across groups at all levels in health and social care services. As such, the complexity of Standards, in an already complex healthcare system adds to challenges with their implementation in health and social care services. Challenges associated with the implementation of complex interventions include variations in “supply side” ^12^ and “demand side.” ^12^ Supply side represents the system and service delivering care. Demand side represents the people using the services and their needs ^12^. Studies that have examined the implementation of health and social care Standards have identified common factors influencing implementation outcomes. For example, an examination of the implementation of the Australian National Safety and Quality in Healthcare Standards (NSQHS) in 2015 identified easily accessible educative materials, stakeholder engagements and credibility as enabling factors to implementing the NSQHS Standards ^13^. A study conducted by NICE in the UK in 2018 aimed at identifying the challenges with implementing the NICE guidance and quality Standards, reported that the main motivating factors to implementation were, improving patient outcomes and local practices ^14^. Themes reflecting the challenges included guidance or Standards not having clear presentation e.g. lengthy documents using medically oriented language, support tools not widely communicated, and evidence not always reflecting ‘real world’ experiences ^14^. Implementation strategies such as educative materials, stakeholder engagements and support tools, as identified in these reports are methods that can leverage enablers to overcome barriers and enhance implementation of an intervention ^15^. Implementation strategies are strongly encouraged and have been described as having “unparalleled importance” in implementation science ^15^. They can comprise single or multi-components. Their main goal is to overcome barriers, help users decipher the intervention and facilitate implementation ^15^. However, limited guidance exists in the literature on appropriate strategies that can act effectively ^16^. In addition, there is a lack of clarity in pairing strategies with stakeholder groups to promote implementation ^16^. This has identified a gap in the literature pertaining to the selection and tailoring of effective support tools that will optimise implementation specifically to health and social care Standards.

Current implementation research addresses components of implementation science in relation to enablers and barriers with specific healthcare interventions or activities including guidelines, evidence-based practices and quality improvement projects. However, while these specific interventions might share some attributes with health and social care Standards e.g. evidence based practice, they do not always demand the multi-level stakeholder buy-in and diverse services that are inherent in Standards. A realist informed review conducted by Dryden-Palmer *et al.* in 2020 investigated context, complexity and processes in the implementation of evidence-based practice ^17^. A total of 67 studies were retrieved, findings relative to context, complexity and process were extracted and grouped into themes and then analysed using a comparative approach. An emergent pattern identified that unsuccessful implementation efforts were linked to a failure to address context. Culture, values and leadership featured under the theme of context and were reported as having positive and negative effects on implementation. In addition, complexity in implementation was strongly linked with a variation in stakeholder roles and accountabilities for the desired intervention change. The authors suggested a focus on improving communication, developing support tools and undergoing implementation on a phased basis as an approach to reduce complexities ^17^. The first step in selecting and tailoring implementation strategies like support tools is to examine factors that will influence implementation ^16^. Factors include characteristics of the intervention, the settings in which the intervention will be implemented and the stakeholders involved in implementation. In addition, such factors that act as enablers in one context may act as barriers in another.

There is a need to examine the literature pertaining to the implementation of health and social care Standards in a global context to capture the multiple stakeholders’ experiences and perceptions and the various contextual aspects associated with the wider audience that Standards apply to. As such, a systematic review will be conducted that seeks to understand not just what factors are influential but why and how they are influential ^12^, thus facilitating a deeper exploration and understanding of the literature findings. A qualitative meta-summary has been chosen to synthesise the available evidence. This mixed research synthesis is deemed an appropriate fit for this review in that it will facilitate collation of the findings from multiple empirical studies carried out in various settings. In addition, it will allow for the identification of viewpoints, be they contradictory or consistent, from a range of stakeholders from both the supply and demand side of health and social care, thus, identifying factors that act as barriers and/or enablers. A comprehensive interpretation of the findings will inform the gaps in current knowledge regarding factors effecting implementation of Standards, and also, inform the design and tailoring of appropriate implementation strategies to enhance their implementation.

A preliminary search of Google Scholar, Cochrane, and EBSCO database did not yield any systematic reviews investigating the enablers and barriers to implementing health and social care Standards. Hence, this review will be the first to identify and describe the enablers and barriers using both qualitative and quantitative research in an integrative synthesis on the implementation of health and social care Standards internationally.

## Protocol

### Research question

What are the enablers and barriers to implementing health and social care Standards in health and social care services?

### Aim

The aim of this protocol is to describe the methodological plan for conducting a systematic review and meta-summary that seeks to identify, describe and synthesise the enablers and barriers that influence implementation of health and social care Standards, from the international literature, in order to inform the development of tailored implementation strategies.

## Methods

This protocol is not eligible for registration with PROSPERO as it is a protocol for a systematic review that seeks to examine aspects of implementation science and will not examine outcomes relevant to clinical or health status. This protocol is reported according to the Preferred Reporting Items for Systematic Reviews and Meta-Analysis Protocols (PRISMA-P) guidelines ^18^ (see reporting guidelines).

Studies for inclusion in this review must fulfil the following criteria:

**Phenomena of interest:** The phenomena of interest from the selected studies will be the identified factors that influence and hinder the implementation of Standards. The term ‘factor’ is defined by the Collins Dictionary as “one of the things that effects an event, decision, or situation.” ^19^ For the purpose of this review, the event or decision or situation refers to the implementation of health and social care Standards. Factors will be categorised under enablers and barriers. The term enabler will be used to refer to any factor that helps to implement Standards more easily. The term barrier will be used to refer to any factor that prevents or hinders the implementation of Standards from happening.

**Type of studies:** Primary research studies that are qualitative, quantitative descriptive and mixed method study designs. Qualitative design studies must use qualitative data collection methods and analysis such as interviews, observations, and thematic analysis. Such studies include ethnography, phenomenology, grounded theory, case studies and qualitative description. Quantitative descriptive studies that will be included are randomised controlled trials, non-randomised controlled trials, cohort studies, case-control studies, cross-sectional studies, prevalence studies, surveys, case series and case reports. Mixed method studies will be included if they use the aforementioned study types and if it’s possible to extract the qualitative and quantitative findings separately from those studies eligible for inclusion.

**Type of participants:** Stakeholders actively involved in health and social care services including patient and public involvement (PPI). Stakeholders will be defined as;

- A person who is employed by a health and/or social care organisation and actively involved in developing and/or implementing health and/or social care Standards. These stakeholders will be categorised according to hierarchical organisational structures from micro (frontline) level, meso (service) level to macro (system) level.
- A person who is a member of the public, which includes a person with “an interest in health and social care as a public service including potential users of services.” ^20^
- A person who uses health and social care services such as “patients, service users, clients or their carers” ^20^

**Type of setting:** All settings where health and social care Standards are implemented.

**Type of interventions:** Studies that examine the implementation of health and social care Standards. Standards refer to quality statements that describe best evidence to achieve quality, safe, and person-centred care. Health and social care Standards are those that are nationally or internationally endorsed. Nationally or internationally endorsed Standards are Standards developed and published by a professional and authoritative organisation and is supported by a local government body.

**Timing and language:** No database time restrictions will be applied. Given the international context of this review, no language limits will be applied. Google translate, university networks or contacting relevant study authors to obtain the English language version of studies are possible sources for language translation if required.

The following will be excluded from this review;

- Studies that examine the implementation of guidelines, policies, protocols, pathways, strategies, guidance, standard operating procedures and Standards that are not nationally or internationally endorsed.
- Educational Standards, Technical Standards, Professional Standards.
- Studies that report secondary data e.g. systematic reviews or scoping reviews. However, the reference lists of any relevant reviews will be screened for potential eligible studies.
- Discussion papers, editorials, opinions, letters, dissertations and conference abstracts.

### Search methods

The bibliographic databases selected for searches are Medline, CINAHL (Cumulative Index to Nursing and Allied Health Literature) and SocINDEX with full text. These databases have been selected to source articles from a broad range of health and social care sciences. The search strategy for this review was formulated using the ‘SPICE’ question framework ^21^ ( Table 1). The concepts of the SPICE framework capture context and stakeholder perspectives ^21, 22^ which are required for a research question seeking to identify enablers and barriers to implementation in health and social care services. Hence the SPICE framework is deemed an appropriate fit for the search strategy. Table 1 displays the SPICE concepts with keywords from the research question. The concept, Comparison (C) is not included, as this was deemed not relevant to the review question in that the aim is not to compare enablers and barriers to implementation of Standards but to extract them from the findings of included studies. The following keywords were included; ‘healthcare’, ‘social care’, ‘Standards’, ‘enabler’, ‘barriers’, ‘implementation.’ Keywords were adapted for searching individual databases, for example using truncation, subject headings and synonyms where applicable. Search terms adapted from the keywords were combined using the Boolean operator ‘OR.’ The fields ‘title’ and ‘abstract’ were searched to identify articles relevant to the research question. The proximity indicator, near operator, ‘N5’ was placed between ‘healthcare’ and ‘Standards’ and also between ‘social care’ and ‘Standards.’ This retrieved studies where these concepts occurred within 5 words of each other. Preliminary searches returned Standards from non-health organisations imposing critical safety measures, for example the aviation industry and World Trade Organisation ^23^. Studies pertaining to Standards in these organisations were considered outside the scope of the research question. The proximity indicator was applied to limit the retrieval of such studies not relevant to health and social care settings that may appear from selected databases given their broad behavioural science inclusion criteria. The search terms from each concept were then connected using the Boolean operator ‘AND.’ Table 2 displays the searches and search returns using the Medline database, as an example.

**Table 1.** SPICE ^*^ framework applied to the research question ^21^.

Setting	Perspectives	Interest, phenomenon of	Comparison (alternate action)	Evaluation
Health and Social Care	Health and Social Care stakeholders, patient and public involvement (PPI)	Enablers and Barriers	Not relevant	Implementation of Standards

*Setting, Perspectives, Interest phenomenon of, Comparison, Evaluation

**Table 2.** Medline database search strings and returns.

Search using EBSCO Interface and Medline database		Search Returns
**Search 1**	Health OR healthcare OR health-care OR "health care" OR "social care" OR "social work" (Ti, AB)	1,997,905
**Search 2**	Standards OR standard (Ti, AB)	1,003,114
**Search 3**	Causes OR influences OR reasons OR determinants OR predictors OR barriers OR obstacles OR challenges OR difficulties OR issues OR problems OR facilitators OR motivators OR enablers OR promoters OR levers OR facilitat* OR enabl* (Ti, AB)	5,545,244
**Search 4**	Implementation OR implementing OR adoption OR acceptance OR adherence OR compliance OR application OR adher* OR implement* OR “use of” OR quality improvement OR (MH “quality improvement”) (Ti, AB)	4,396,731
**Search 5**	S1 **N5** S2	14,973
**Search 6**	S5 AND S3 AND S4	2,859

MH: Mesh Headings Ti, AB: Titles, Abstracts

In addition, a sensitivity analysis was conducted to ensure that key papers were not lost when using the proximity indicator. Two studies had been identified through preliminary hand searching of the literature and they both examined the implementation of healthcare Standards. As such, these papers were used to test the sensitivity of the final search returns which included the use of the proximity indicator, in which case both papers were retrieved from the search ^13, 24^.

Two reviewers will independently screen titles and abstracts based on inclusion and exclusion criteria. Agreement on the studies for inclusion will be reached and any uncertainties will be discussed and resolved. If consensus cannot be reached on studies eligible for inclusion, a third independent reviewer will be invited to screen titles and abstracts. The full text of the studies that are identified in the screening of titles and abstracts will be read independently by two reviewers to confirm that they fulfil the inclusion criteria as defined in the methods section. Again, if agreement cannot be reached on studies fulfilling the inclusion criteria, a third independent reviewer will be asked to read the full text studies selected for inclusion and then decisions will be agreed based on consensus between the three reviewers.

Health and social care Standards are most commonly developed and published by Standards-setting bodies. It is expected that relevant studies for this review may also be sourced from the grey literature. Grey literature can be described as documents that are not formally published in sources such as academic journals or easily accessible databases ^25^. Rigorous systematic methods to conducting grey literature searches are scarce ^26^. A search method used by Godin *et al*. (2015) ^26^ will be adopted to retrieve potential studies for inclusion from the grey literature. This method offers a systematic approach and begins with developing a grey literature search plan ^26^. The search plan will comprise two steps; 1. Grey literature databases and 2. Targeted website searches of Standards-setting bodies.

**Step 1:** Google Scholar, OpenGrey and GreyNet International are the grey literature databases that will be used in this review. Keywords from the review question will be adapted to fit these databases. Keywords will include; ‘healthcare’, ‘social care’, ‘Standards’ and ‘implementation’. The titles of the search returns will be reviewed for eligibility and studies deemed potentially relevant to the review question will be highlighted for further review. For each database search, a record of keywords used, search returns and studies reviewed for eligibility will be recorded.

**Step 2:** The second search method will be searching targeted websites of Standards-setting bodies relevant to health and social care. Godin *et al.* described this approach as being similar to a hand-searching method ^26^. The researcher will identify Standards-setting bodies from a review conducted by the Standard-setting body in Ireland, the Health Information and Quality Authority (HIQA) ^27^. This review examined how international Standards-setting bodies develop Standards and guidance for health and social care services and included 13 organisations from nine jurisdictions ^27^. The Google Chrome search engine and manual searches will be used to access the websites belonging to these Standards-setting bodies. Keywords used in step 1 (‘healthcare’, ‘social care’, ‘Standards’ and ‘implementation’) will be applied to the search bar function on the website’s homepage. If the search functionality does not exist, hand searching the website’s homepage will be conducted to retrieve documents relevant to the review question. Filters will not be applied to the searches.

Given the potential for high volumes of search returns in step one and step two, the research team will review the titles and abstracts of studies retrieved on the first 10 pages of the search or the first 100 hits. It is possible in grey literature that studies may not have abstracts and so, any study without an abstract that is deemed potentially relevant, from its title, will be read in full text to ascertain eligibility. A record of the searching process will be kept including date and time of searches, listing names of Standards-setting bodies and their website addresses (URLs).

An additional “good practice” ^22^ approach will be taken as described by Booth in 2016. The context of references used within the included studies, along with their reference lists will be hand searched to check for eligible inclusion in this review ^22^.

The results from the bibliographic database and grey literature search, screening and outcomes will be displayed using the flow diagram as recommended by the Preferred Reporting Items for Systematic Reviews (PRISMA) ^28^. Reasons for exclusion of studies at full text review will be recorded.

### Data management

Selected articles will be stored and managed using EndNote ^TM^ X8.2 Reference Manager Library. The search results will be imported into the online Covidence systematic management system. Covidence will also be used to facilitate the sharing and collaboration between reviewers during the screening of abstracts and titles, data extraction and quality appraisal stages.

### Data extraction

A data extraction table will be populated to structure and categorise the findings (see extended data ^29^). Microsoft Excel and Covidence will be used to manage and store the extracted data. Data items from selected studies that are extracted will be populated using two tables. The first table will have two sections; 1. General Information - first author name, year of publication, origin of study location, 2. Study Design (methods) - setting, intervention (title of Standards), aim of study, sample population, sample size, data collection method, and analytical approach. The second table will categorise the study outcomes from the primary studies under reported enablers and barriers to implementing health and social care Standards. This will comprise second order constructs. Second order constructs are described by Butler *et al.* (2016) as the researcher’s descriptions, discussions, interpretations, statements and ideas ^30^.

Two reviewers will independently extract these data. The data extraction tables will be piloted on four studies before its application to the remainder of the studies. In addition, every effort to retain the original content and context of the selected studies will be made. Any disagreements or discrepancies with extracted data will be discussed and resolved. If consensus is not reached, a third reviewer will be asked to independently check these data for accuracy and resolution of any disagreements.

### Quality assessment

This review will apply the following critical appraisal tools to assess the methodological quality of selected studies; Critical Appraisal Skills Programme (CASP) tools will be used for qualitative studies ^31^, Joanna Briggs Institute Critical Appraisal Tools will be used for quantitative studies ^32^, and The Mixed Methods Appraisal Tool (MMAT) ^33^ will be used for mixed method studies. 

Two researchers will independently appraise the quality of the selected studies. Any discrepancies with study assessments will be discussed and resolved. If agreement cannot be reached, a third researcher will be asked to appraise the studies to come to a consensus. Studies will not be excluded based on quality of evidence.

### Data synthesis

**Figure 1.**
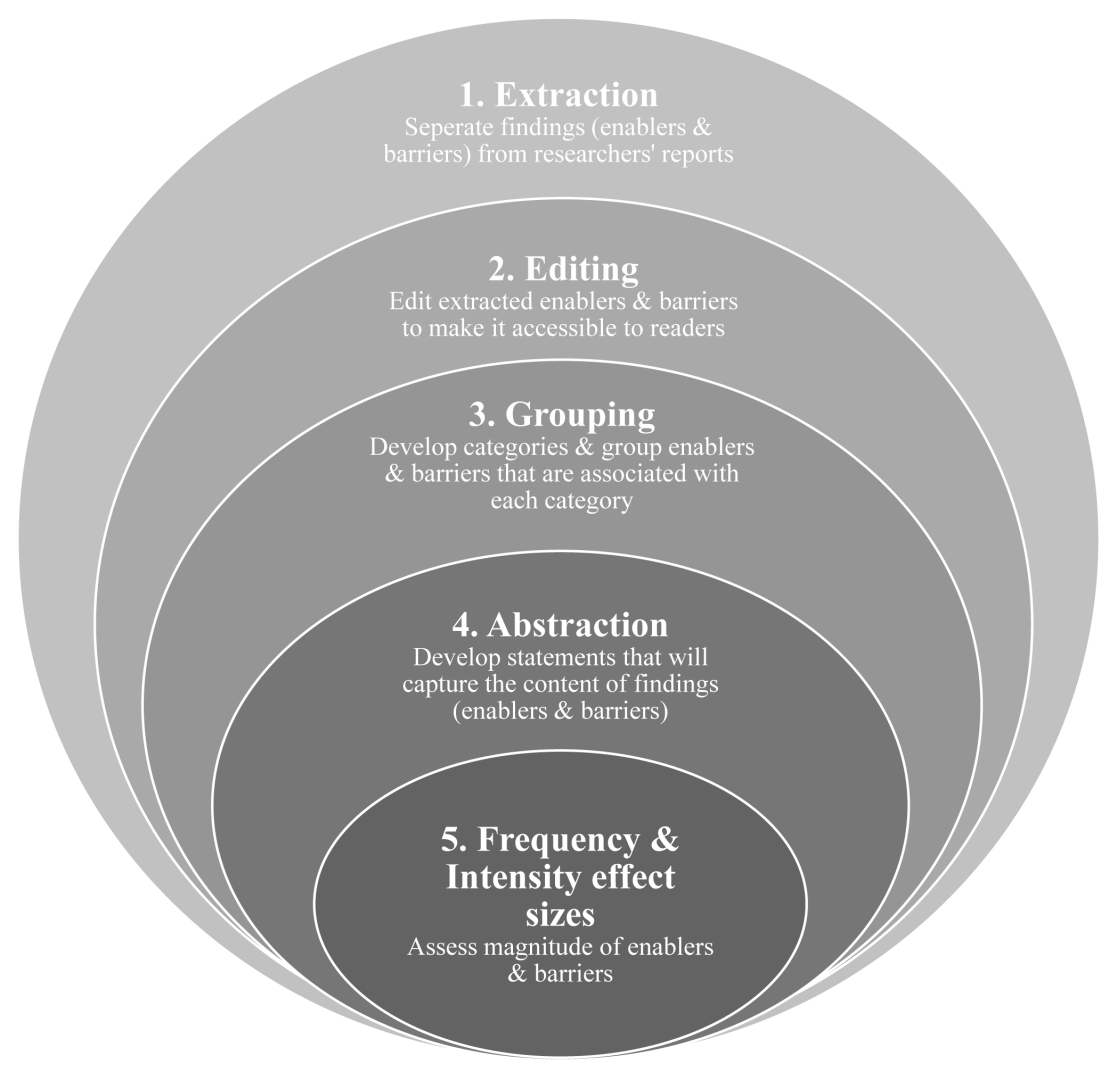
Five steps of meta-summary adapted from Sandelowski and Barroso (2007) ^34^.

Qualitative meta-summary will be used to synthesise the descriptive findings from qualitative and quantitative studies. As such, it will be a mixed research method synthesis that will aggregate and integrate the findings from the included studies ^34^. This will facilitate a deeper understanding and evaluation of each theme identified as an enabler or barrier. Meta-summary was developed by Sandelowski and Barroso (2007) and comprises a five step technique that provides a quantitative element to represent the findings ^35^. The quantitative element is reflected in effect sizes that measure how often (frequency) the enablers and barriers are reported in the studies and how strong (intensity) the reported enablers and barriers are among the studies. The peeling of the onion metaphor is used to conceptualise each step involved in a meta-summary. Each layer of the onion represents each step in which the data is carefully unpacked or peeled away to reach the core which is the effect sizes of each enabler and barrier ( Figure 1).

One researcher will conduct the data analysis and will discuss findings with a senior researcher to ensure that the extracted data appropriately reflects the primary data. This will enhance transparency, replicability and trustworthiness of the findings ^30^.

### Assessment of confidence in evidence

The GRADE-CERQual (Grades of Recommendation, Assessment, Development, and Evaluation-Confidence in Evidence from Reviews of Qualitative research) approach will be used to assess confidence in the qualitative evidence synthesis ^37^. The outcome of interest will be confidence in the evidence for the identified enablers and barriers to implementing health and social care Standards. The assessment will be based on four domains which are methodological limitations of primary studies used in the synthesis, the relevance of the primary studies with regard to the review question, the coherence of the findings from the primary studies and the adequacy of the data supporting the findings ^37^. This will be rated as high, moderate, low or very low and a reason will be provided for a given judgement. These assessments will be undertaken independently by two researchers and once agreement is reached, a summary of findings table will be prepared.

### Ethics and dissemination

The research does not require ethical approval due to its retrospective nature and no involvement of persons in the study. The dissemination strategy will include presentations of the research findings at conferences and publishing in an open access peer reviewed journal.

### Study status

The bibliographic database search was conducted in November 2020. Screening of titles and abstracts was completed in January 2021. Full-text screening has commenced. It is anticipated that the grey literature search will commence in February 2021.

### Strengths and limitations

This review will be the first to systematically examine reported enablers and barriers to implementing health and social care Standards in a global context. A strength is that we plan to include literature from varied study designs, health and social care settings and stakeholders, to reflect the wide systemic nature of health and social care Standards in practice. The mixed research synthesis using the Sandelowki and Barroso meta-summary ^34^ will facilitate the synthesis of findings from different methodological approaches. This will result in a comprehensive examination of factors identified as enablers and barriers to implementing health and social care Standards and quantify their prevalence across the literature. In addition, the use of the validated tool, GRADE-CERQual to assess confidence in study findings will indicate quality and credibility in the identified enablers and barriers.

The inherent lack of structure and disparate sources associated with grey literature may hinder the retrieval of all relevant studies for this review. Conversely, the inclusion of a grey literature search and targeted website searches, will complement the search of academic publication databases and contribute to a comprehensive search of the literature.

## Conclusion

This protocol describes the methodological steps in conducting a systematic review to identify, describe and synthesise factors that influence or hinder implementation of health and social care Standards. The rigorous approach to searching the literature, appraising the selected studies, data extraction and a mixed research synthesis described herein, will enhance the interpretation and usability of the research findings. The use of meta-summary to calculate the effect sizes of identified enablers and barriers will facilitate evaluation of the magnitude and concentration of such factors across the literature, giving weight to their potential impact when included in implementation strategies. The findings from this review will be valuable to stakeholders who develop and implement health and social care Standards in health and social care services. In addition, the identification of enablers and barriers will inform the development and tailoring of tools to support implementation of Standards in practice.

## Data availability

### Underlying data

No data are associated with this article.

### Extended data

Figshare: Data Extraction Tables: Systematic Review and Metasummary YK 2020.docx. https://doi.org/10.6084/m9.figshare.13289201.v2 ^29^

This protocol contains the following extended data:

- Data Extraction Tables SR Metasummary YK 2021.docx (Data extraction table)

### Reporting guidelines

Repository: PRISMA-P checklist for Factors that influence the implementation of health and social care Standards: a systematic review and meta-summary protocol. https://doi.org/10.6084/m9.figshare.13289138.v2 ^38^

Data are available under the terms of the Creative Commons Attribution 4.0 International license (CC-BY 4.0).
