# Supplementary material for: Factors that influence the implementation of health and social care Standards: a systematic review and meta-summary protocol
Source: HRB Open Res. 2021 May 24;4:24. Originally published 2021 Feb 24. [Version 2] doi: 10.12688/hrbopenres.13212.2 (PMC8278248; doi:10.12688/hrbopenres.13212.2)
Supplement: Peer review history

# Peer review: Reviewer response for version 2

**DOI:** 10.21956/hrbopenres.14497.r29700

**Published:** 2021-07-13

**Authors:** Faiza Anjum

**Affiliations:** 1 Sociology, National Textile University, Faisalabad, Pakistan

I am pleased to inform that this paper of a systematic review of factors influencing the implementation of health and social care standards and its meta-summary protocol is interesting. The authors have a deep insight into identifying a gap in the review of the literature. The study has a comprehensive analysis through addressing quantitative, qualitative, and mixed methods.

   

 The rationale of the study and objectives of the study are clearly described, contributing a valuable addition to the existing literature. The references of the study are best suited to the nature of the study. The adopted methods/design are also appropriate for the study question.

 However, the authors have not described the recommendation for further study in the future. In a comprehensive analysis, there is a need to add a theoretical background in a separate paragraph.

 I confirm that I have reviewed the article and suggested that there is no extensive need for revision for indexing.

Is the study design appropriate for the research question?

Yes

Is the rationale for, and objectives of, the study clearly described?

Yes

Are sufficient details of the methods provided to allow replication by others?

Yes

Are the datasets clearly presented in a useable and accessible format?

Yes

Reviewer Expertise:

Sociology, Medical Sociology, Social psychology, Environment and society, Women health issues

I confirm that I have read this submission and believe that I have an appropriate level of expertise to confirm that it is of an acceptable scientific standard.

# Peer review: Reviewer response for version 1

**DOI:** 10.21956/hrbopenres.14364.r29212

**Published:** 2021-04-21

**Authors:** Harriet Boulding

**Affiliations:** 1 The Policy Institute, King's College London, London, UK

I was pleased to review this protocol for a systematic review of barriers and facilitators to implementing health and social care standards. The authors have identified a gap in the review literature and have put forward a comprehensive proposal to address this, which includes qualitative, quantitative and mixed methods studies, and, importantly, a search of grey literature. The authors propose to highlight the experiences and perspectives of a range of stakeholders, including health service employees at various levels, patients and the public. The findings are intended to inform the development and implementation of health and social care standards, and the development of tools to support implementation.

 The rationale for the study is clearly described, and such work would be a timely addition to the literature. The authors have referenced appropriate standards for reporting, quality assessment, data management, and search methods. The study design is appropriate for the research question, though the authors will need to clearly explain their approach re the question framework in terms of comparison v identification. The focus on a range of stakeholder perspectives is particularly welcome. I am happy to recommend the protocol for indexing with the following advice to the authors:

- The review is global in nature, but there is no discussion of the very distinct contexts and implementation challenges in LMICs, or the relevance of this for search methods/sources. More is needed to highlight the benefits and challenges of extracting useful lessons for implementation from such disparate contexts.
- On a related note, I would welcome a clearer and more consistent discussion of the study strengths and objectives, as these appear to be a little different in the abstract, background and conclusion.
- More clarity on inclusion and exclusion criteria is needed in several areas. There should be some parity between the types of qual and quant methods included, whereas we currently have a comprehensive list of the types of quant studies, and some examples of qual studies. The rationale for the inclusion and exclusion criteria should be stated clearly throughout.
- The inclusion of grey literature is of particular importance for work examining implementation in healthcare settings. The authors have acknowledged this indirectly via their study design, but I would welcome a discussion of this element in their background discussion given its relevance to this endeavour.
- I note that HMIC (health management information consortium) database has not been included; it may yield some pertinent results not captured elsewhere, particularly grey literature, if possible to examine at this stage.

Thank you for the opportunity to review this important work, and I look forward to seeing the protocol and the subsequent review in print.

Is the study design appropriate for the research question?

Yes

Is the rationale for, and objectives of, the study clearly described?

Yes

Are sufficient details of the methods provided to allow replication by others?

Yes

Are the datasets clearly presented in a useable and accessible format?

Yes

Reviewer Expertise:

Health systems, policy, implementation.

I confirm that I have read this submission and believe that I have an appropriate level of expertise to confirm that it is of an acceptable scientific standard.
